# A Novel Electrochemical Sensor Based on Ti_3_C_2_T_x_ MXene/Mesoporous Hollow Carbon Sphere Hybrid to Detect Bisphenol A

**DOI:** 10.3390/molecules30193992

**Published:** 2025-10-05

**Authors:** Fei Cao, Qirong Zhou, Yanting Zhou, Yaqi Yang, Li Zhang, Yixi Xie

**Affiliations:** 1 College of Chemistry and Materials Engineering, Huaihua University, Huaihua 418000, China; 18873717089@163.com (F.C.); 18874532577@163.com (Q.Z.); zhou168544378@163.com (Y.Z.); 2 Key Laboratory for Green Organic Synthesis and Application of Hunan Province, Xiangtan University, Xiangtan 411105, China

**Keywords:** Ti_3_C_2_T_x_ MXene, MHCs, BPA, electrochemical sensors

## Abstract

Bisphenol A (2,2-bis(4-hydroxyphenyl)propane, BPA), an endocrine-disrupting chemical with recognized adverse effects on human health and ecosystems, urgently requires convenient, sensitive, and accurate detection methods. In this study, a hierarchical heterostructure was fabricated by incorporating Ti_3_C_2_T_x_ MXene and mesoporous hollow carbon spheres (MHCs) to develop a high-performance electrochemical sensor for BPA. The nanocomposite was thoroughly characterized using SEM, TEM, and XRD, and then applied to modify a glassy carbon electrode (GCE). Under optimized conditions including pH and accumulation time, BPA detection was carried out via differential pulse voltammetry (DPV). The sensor exhibited a wide linear detection range from 10 to 200 μM and a low detection limit of 2.6 μM. Moreover, it was successfully applied to environmental water samples, demonstrating high accuracy and practicality for real-world BPA monitoring.

## 1. Introduction

Bisphenol A (BPA), a well-documented endocrine-disrupting chemical (EDC), exerts estrogen-mimicking effects that interfere with the normal function of the endocrine system, leading to severe adverse outcomes in reproduction, development, and metabolism [1,2,3,4]. A growing body of epidemiological evidence has linked elevated human exposure to BPA with an increased incidence of various serious diseases, including but not limited to certain cancers, type 2 diabetes, thyroid dysfunction, and declining sperm quality [5,6,7]. Despite these recognized health risks, BPA remains a cornerstone in the manufacturing of polycarbonate plastics and epoxy resins due to its exceptional physicochemical properties, such as high durability, optical clarity, and thermal stability. This has led to its pervasive use in a wide array of daily consumer products, from reusable water bottles and food storage containers to the protective linings of beverage cans [8,9,10,11]. Consequently, BPA has become a ubiquitous environmental contaminant that is consistently detected in water sources, soil, dust, and, importantly, in human biological samples such as urine and blood, confirming systemic exposure [12,13]. The potential for bioaccumulation and the irreversible damage it can cause to both human and ecosystem health upon chronic exposure underscore the critical necessity for robust monitoring techniques.

To address this need, a variety of analytical methods have been employed for BPA detection. Conventional techniques, such as high-performance liquid chromatography (HPLC) coupled with mass spectrometry [14], chemiluminescent immunoassays [15], and enzyme-linked immunosorbent assays (ELISA) [16], are renowned for their high sensitivity and accuracy, often serving as gold standards for laboratory confirmation. However, these methods are frequently constrained by their reliance on sophisticated costly instrumentation, the need for skilled operators, time-consuming sample preparation procedures, and limited suitability for rapid on-site analysis. In contrast, electrochemical sensors have emerged as a powerful and promising alternative [4,12,17]. They offer a compelling combination of advantages: rapid response, low cost, simple operation, and excellent potential for miniaturization and integration into portable devices for continuous in-field monitoring. While significant progress has been made, the development of novel electrode materials with enhanced sensitivity, selectivity, and anti-fouling properties remains a vibrant area of research, aiming to overcome limitations of existing sensor platforms and fully realize the potential of electrochemical techniques for decentralized BPA detection.

MXene, a class of two-dimensional layered materials, is typically synthesized by selectively etching the A atomic layer (e.g., aluminum) from the precursor MAX phase. This process yields layered structures with rich surface functional groups (such as -O, -OH, -F), which contribute to their high electrical conductivity, substantial specific surface area, and excellent hydrophilicity. As a result, MXenes have attracted significant attention and are widely employed in energy storage devices, supercapacitors, and electrochemical sensors [18,19,20,21]. However, the practical application of MXene is often limited by its susceptibility to oxidation and the tendency of its layers to restack, which reduce active surface area and degrade electrochemical performance. To address these issues, one effective strategy is to incorporate other functional materials into MXene layers to form composite structures. For instance, Ti_3_C_2_T_x_@rGO composites exhibit enhanced specific capacitance and mechanical stability, making them highly suitable for supercapacitor applications [22]. Similarly, alk-Ti_3_C_2_/Co-Zn-NC hybrid materials have been utilized to construct sensitive electrochemical sensors for detecting 4-nitrophenol [20].

Among various supporting materials, mesoporous hollow carbon spheres (MHCs) have garnered widespread interest due to their unique structural and chemical properties, including high specific surface area, tunable pore structure, excellent chemical stability, and favorable electrical conductivity [23,24,25,26,27]. The integration of MHCs with MXene not only mitigates layer restacking, but also further increases the specific surface area and enhances electron transfer within the composite. Moreover, the porous carbon framework can serve as a physical barrier that improves the oxidation stability of MXene. Consequently, MXene/carbon composites combine the advantages of both components, resulting in superior electrical conductivity, structural durability, and electrochemical activity. These enhancements make such composite materials promising candidates for a broader range of advanced applications, from enhanced energy storage systems to high-performance sensors.

**Scheme 1:**
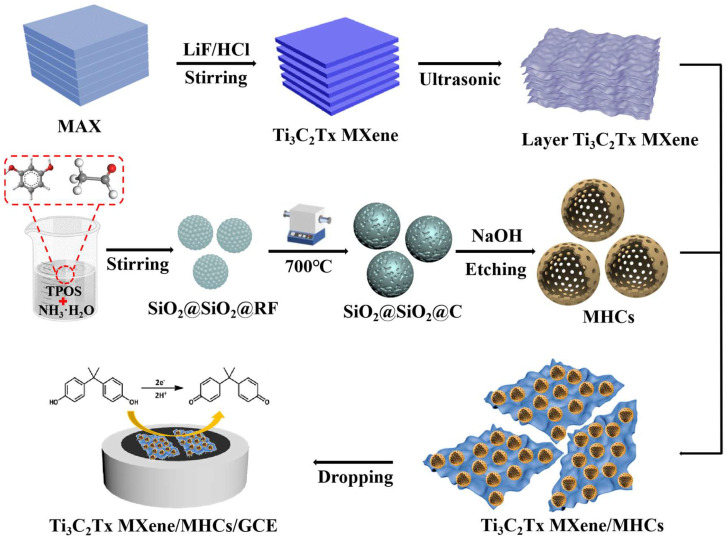
Schematic representation of the fabrication procedure of MXene/MHCs/GCE.

Herein, the accordion-like layered structure of Ti_3_C_2_T_x_ MXene was successfully synthesized via an in situ lithium fluoride and hydrochloric acid (LiF/HCl) etching method from the MAX phase (Ti_3_AlC_2_). This unique architecture provides a foundational high surface area and conductivity. To address the inherent tendency of MXene layers to restack, which severely compromises their active surface area and ion accessibility, mesoporous hollow carbon spheres (MHCs) were introduced as nano-spacers. The incorporation of MHCs effectively inhibits the restacking of MXene sheets, thereby preserving the interlayer spacing and facilitating electrolyte penetration. More importantly, this strategic combination creates a synergistic effect: the MHCs not only provide abundant surface pores and shorten the ion diffusion pathways, but also enhance the overall conductivity by acting as conductive bridges between the MXene layers. Consequently, the electron/ion transport kinetics within the composite are significantly accelerated. Furthermore, the robust carbon framework of MHCs reinforces the structural stability of the composite, preventing the degradation of MXene while maintaining its original sheet-like morphology. Owing to these attributes—exceptional electrical conductivity, a large accessible surface area, and hierarchical porous channels—the Ti_3_C_2_T_x_ MXene/MHCs composite was employed to fabricate an electrochemical sensor for BPA (Scheme 1). The resulting sensor exhibited remarkable electrochemical performance, enabling the highly sensitive and stable detection of BPA.

## 2. Results and Discussion

### 2.1. Characterization

**Figure 1:**
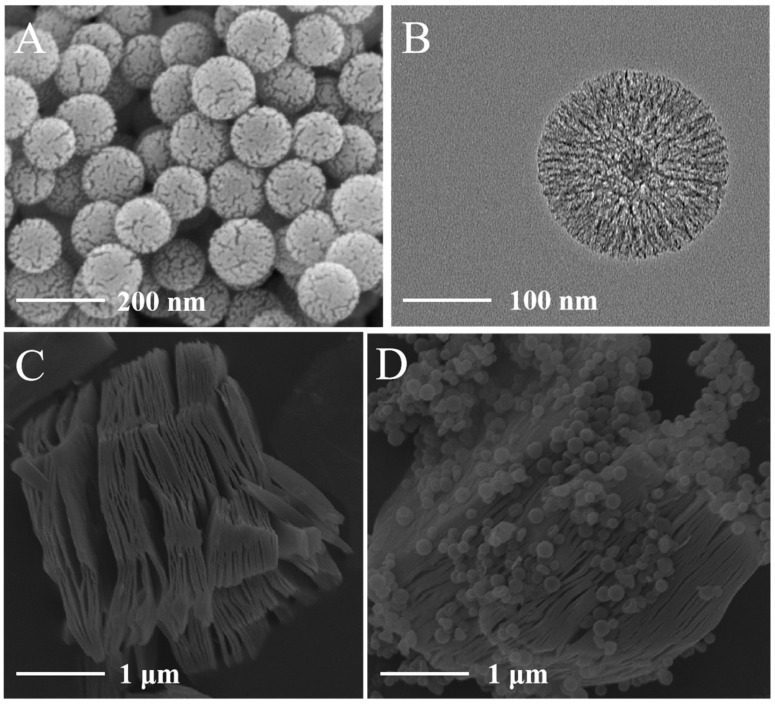
The SEM images of (**A**) MHCs, (**C**) MXene, and (**D**) MXene/MHCs. (**B**) The TEM image of MHCs.

The synthesis of the hierarchical composite involved a multi-step process, beginning with the preparation of spherical SiO_2_@SiO_2_@RF particles via a modified Stöber method combined with resorcinol-formaldehyde (RF) resin coating. As shown in Figure S1A, the as-synthesized particles exhibited a well-defined uniform spherical morphology with a smooth surface. Subsequent high-temperature pyrolysis under an inert atmosphere carbonized the RF resin shell into a porous carbon layer. This transformation was evidenced by a distinct change in the surface texture from smooth to rough (Figure S1B), attributable to the decomposition of organic components and the formation of a carbon framework, while the overall spherical architecture remained intact. The critical step of creating the hollow structure was achieved through etching with a strong alkaline solution (NaOH). This treatment selectively dissolved the inner silica templates, leaving the mesoporous carbon shell. Remarkably, the spherical morphology was preserved even after this etching process, with the surface displaying pronounced etching pits and a highly porous structure under SEM observation (Figure 1A). The successful formation of the hollow interior was unambiguously confirmed by transmission electron microscopy (TEM) (Figure 1B), which revealed a clear contrast between the dark shell and the bright cavity, identifying the final product as mesoporous hollow carbon spheres (MHCs).

In a parallel procedure, the accordion-like Ti_3_C_2_T_x_ MXene was synthesized by etching the Ti_3_AlC_2_ (MAX phase) precursor with a LiF/HCl solution. This process selectively removed the Al layers, resulting in a multi-layered sheet-like material with a characteristic loosely stacked morphology, as clearly observed in Figure 1C. Finally, the Ti_3_C_2_T_x_ MXene/MHCs composite was constructed by a simple ultrasonic-assisted self-assembly method. As depicted in Figure 1D, the MHCs were uniformly dispersed and firmly anchored onto the surface of the MXene sheets. This intimate contact is crucial for preventing the restacking of MXene layers and facilitating synergistic effects in the composite material.

Subsequently, the crystal structure of MHCs, Ti_3_AlC_2_, and Ti_3_C_2_T_x_ MXene were investigated by XRD. As shown in Figure S2A, diffraction peaks appear at around 2θ = 25°, corresponding to the C (002) plane. Simultaneously, the distinct diffraction peaks of 2θ = 19.2°, 33.9°, 36.6°, 38.8°, 41.7°, 44.7°, 48.2°, 56.3°, and 60.1° corresponded to the (004), (101), (103), (104), (105), (106), (107), (109), and (110) planes of MAX [28]. Part of the Al layer in Ti_3_AlC_2_ was selectively removed after HCl/LiF etching. The characteristic peak (104) of Ti_3_AlC_2_ at 39.6° is significantly attenuated by the selective removal of the Al atomic layer. Diffraction peaks at 29.6° and 61.2° corresponded to the (006) and (110) planes of Ti_3_C_2_T_x_, indicating the Ti_3_C_2_T_x_ MXene was successfully obtained [29].

XPS was used to characterize the elemental composition and chemical state of SiO_2_@SiO_2_@C and MHCs. C and O were found in MHCs (Figure S2B). However, the Si element only exists in SiO_2_@SiO_2_@C. After strong alkali etching, it can be observed from the figure that the Si element has been removed and the C element content has significantly increased. As a result, uniform hollow mesoporous beads with excellent conductivity were produced to facilitate the subsequent construction of electrochemical sensors designed to detect BPA. Furthermore, the nitrogen adsorption–desorption isotherms are depicted in Figure S2C,D. The Barrett–Joyner–Halenda (BJH) pore size distribution is depicted in the figure as an inset. These isotherms of adsorption and desorption are typical IV curves found in mesoporous materials. The specific surface area and pore volume are raised to 1718.48 m^2^/g and 1.898 m^3^/g, respectively.

### 2.2. Electrochemical Characterization of BPA on Different Electrodes

**Figure 2:**
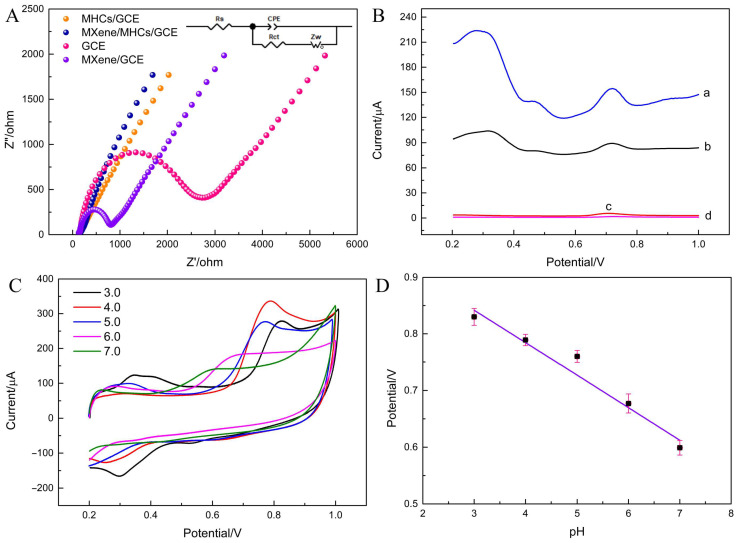
(**A**) The Nyquist plots of modified electrodes; supporting electrolyte: 0.1 M KCl containing 5 mM Fe(CN)_6_^3−/4−^; frequency range: 0.01 Hz to 100 KHz. (**B**) DPVs of 100 μM BPA on bare GCE (d), MXene/GCE(c), MHCs/GCE (b) and MXene/MHCs/GCE (a) in 0.1 M PBS (pH = 4.0). (**C**) CV curves of MXene/MHCs/GCE at different pH values (3.0, 4.0, 5.0, 6.0, 7.0) in 0.1 M PBS containing 500 μM BPA. (**D**) Plots of Epa versus pH value for BPA.

Electrochemical impedance spectroscopy (EIS) was employed to evaluate the electron transfer resistance (Rct) of different electrodes: bare GCE (Rct = 2645.8 Ω), Ti_3_C_2_T_x_ MXene/GCE (Rct = 791.2 Ω), MHCs/GCE (Rct = 139.3 Ω), and Ti_3_C_2_T_x_ MXene/MHCs/GCE (Rct = 275.1 Ω) (Figure 2A). The Nyquist plot revealed that the bare GCE exhibited the largest semicircular diameter, suggesting the highest electron transfer resistance. In contrast, both the MHCs and Ti_3_C_2_T_x_ MXene/MHCs modified electrodes showed significantly diminished semicircles that were nearly linear, indicating markedly lower charge transfer resistance. These results demonstrate that the materials derived from pyrolysis and alkali treatment, especially when combined with Ti_3_C_2_T_x_ MXene, facilitate electron transfer substantially and enhance overall electrical conductivity.

Compared with Bare/GCE (0.91 μA), Ti_3_C_2_T_x_ MXene/GCE (2.28 μA), and MHCs/GCE (4.97 μA), Ti_3_C_2_T_x_ MXene/MHCs/GCE (16.89 μA) acquired the most peak current of BPA oxidation, indicating that the Ti_3_C_2_T_x_ MXene/MHCs composite material not only avoided the problem of restacking of two-dimensional nanomaterials (Ti_3_C_2_T_x_ MXene), but also enhanced the abundant surface pores and good conductivity of MHCs, improved the permeability of electrolyte solution, and shortened the ion transport path (Figure 2B).

### 2.3. Optimization of Material Ratio

The electrode modified with composite materials composed of different proportions of materials has a certain impact on the detection of BPA. Therefore, we investigated the composition of materials Ti_3_C_2_T_x_ MXene and MHCs. As shown in Figure S3C, we selected different ratios of MHCs/Ti_3_C_2_T_x_ MXene (1:2, 2:3, 1:1). As the proportion of the MHCs material increased, that is, when the ratio reached 2:3, the maximum peak current value was obtained for detecting BPA. At this ratio, the obtained composite material exhibits the best detection performance for BPA. Therefore, we choose 2:3 as the optimal ratio.

### 2.4. Optimum Determination Conditions

The influence of pH (ranging from 3.0 to 7.0) on the electrochemical response of BPA at the Ti_3_C_2_T_x_ MXene/MHCs/GCE was investigated. The peak current (ipa) varied with pH, reaching its maximum at pH 4.0, which was, therefore, selected as the optimal value. Additionally, the anodic peak potential (Epa) shifted negatively with increasing pH, suggesting the involvement of protons in the electrode reaction (Figure 2C,D).

Epa (BPA, V) = −0.0574 pH + 1.014 (R^2^ = 0.9013)

Of these, the slope of BPA is −0.0574. This is approximately −0.0591 V/pH (Figure 2D). According to Nernst equation [30], the following equation can be obtained.

$$\frac{dEp}{dpH}=\frac{2.303mRT}{nF}$$

Of these, m/n = 1, meaning the BPA on Ti_3_C_2_T_x_ MXene/MHCs/GCE is an isoelectric–isoprotic process. The scheme of BPA electro-oxidation at Ti_3_C_2_T_x_ MXene/MHCs/GCE is presented in Scheme S1.

The strong adsorption capacity of Ti_3_C_2_T_x_ MXene/MHCs/GCE for BPA necessitates the optimization of both accumulation potential (−0.3 to 0.2 V) and accumulation time (0 to 120 s), as these parameters govern the detection performance of BPA. The effect of enrichment time on BPA detection was investigated (Figure S3A). The peak current (ipa) increased with prolonged enrichment time until reaching a maximum at 60 s, beyond which it remained nearly constant. Therefore, 60 s was chosen as the optimal enrichment time. Additionally, the influence of enrichment potential was examined over the range from −0.3 V to −0.2 V (Figure S3B). The highest ipa was achieved at −0.2 V, which was selected as the optimum potential for subsequent experiments.

### 2.5. Electrochemical Behavior of Ti_3_C_2_T_x_ MXene/MHCs/GCE at Different Scan Rates

**Figure 3:**
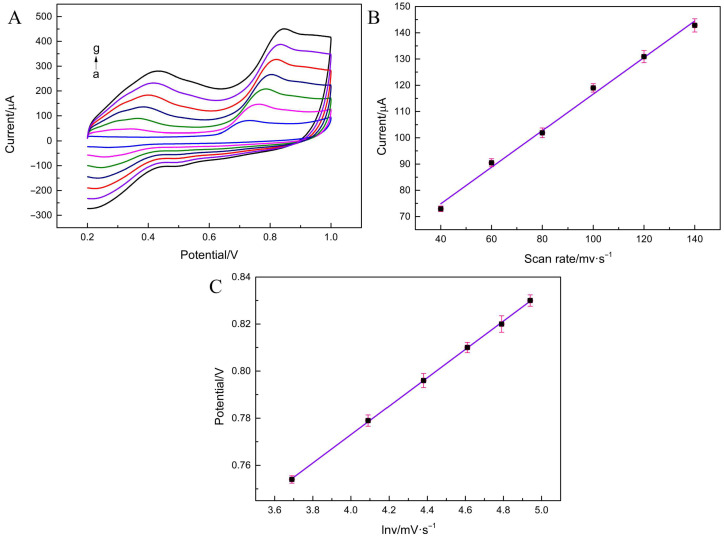
(**A**) CV curves of MXene/MHCs/GCE at different scan rates in 0.1 M PBS (pH = 4.0) containing 500 μM BPA. (**B**) The dependence of redox current on the scan rate. (**C**) The dependence of redox potential on lnν.

To investigate the performance of BPA in the oxidation mechanism on Ti_3_C_2_T_x_ MXene/MHCs/GCE, the scanning rates were examined (Figure 3). In this case, both v and ipa showed the same trend, with ipa correlating with v (Figure 3C), indicating that the process is adsorption-controlled.

ipa (BPA, μA) = 0.6965 v + 46.99 (R^2^ = 0.9942)

In the meantime, the irreversibility of the process of BPA on Ti_3_C_2_T_x_ MXene/MHCs/GCE is indicated by the the Ep/V relationship.

Epa (BPA) = 0.0603 ln v (mV/s) + 0.5318 (R^2^ = 0.9997)

Using Laviron’s equation [31], we obtained the following.

$$Epa\left(V\right)=E^{\theta }+(\frac{RT}{\alpha n_{\alpha }F})\;\mathrm{lg}\;(\frac{RTk^{\theta }}{\alpha n_{\alpha }F})+\left(\frac{RT}{\alpha n_{\alpha }F}\right)lnv$$

Of these, R, F, and T remain constant. From the above formula, the range for α is approximately 0.4–0.7. Therefore, α is taken as 0.426, and n is 2. Thus, the BPA redox process involves two electron transfers during the whole oxidation process.

### 2.6. Determination of BPA on Ti_3_C_2_T_x_ MXene/MHCs/GCE

**Figure 4:**
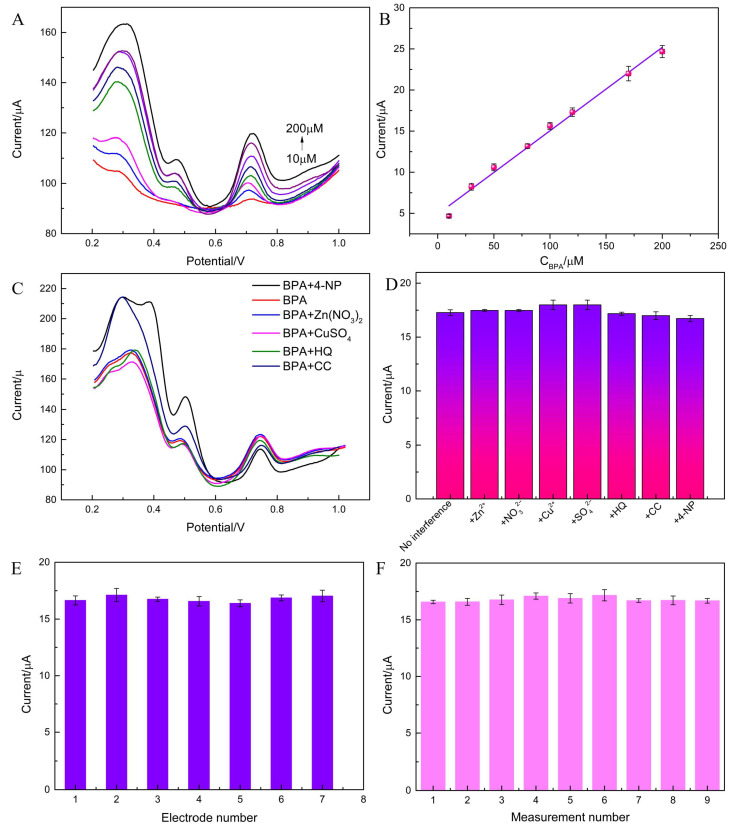
(**A**) DPVs obtained from MXene/MHCs/GCE in the presence of BPA ranging from a to h (10, 30, 50, 80, 100, 120, 170, 200 μmol/L) in 0.1M PBS (pH 4.0). (**B**) The DPVs of linear calibration between the current responses and the concentration of BPA. (**C**,**D**) Effect of some possible interfering substances on the determination of 100 μM BPA in PBS solution. (**E**,**F**) Reproducibility study of seven individual electrodes in the same conditions.

Optimal concentrations and peak currents were obtained. Within some concentration (BPA: 10–200 μM), the corresponding ipas increase with increasing concentration and show a certain linear relationship (Figure 4A,B).

ipa (BPA, μA) = 0.1014 C (μM) + 4.899 (10 μM ≤ C ≤ 200 μM, R^2^ = 0.9916)

The LOD was 2.6 μM (S/N = 3). Ti_3_C_2_T_x_ MXene/MHCs/GCE provided the sensitive detection of BPA (Table 1).

### 2.7. Stability, Reproducibility, and Anti-Interference

Firstly, ipa was acquired from parallel experiments and GCE was decorated with Ti_3_C_2_T_x_ MXene/MHCs/GCE, which did not show significant changes, and with corresponding RSDs of 1.3% and 0.89%, respectively (Figure 4E,F). The addition of 50 times more ions (Zn^2+^, Cu^2+^, SO_4_^2−^, NO_3_^−^) and 10 times more hydroquinone (HQ), catechol (CC), and 4-nitrophenol (4-NP) to PBS (100 μM BPA) one by one did not cause any significant change in ipa (Figure 4C,D). The stability of Ti_3_C_2_T_x_ MXene/MHCs/GCE was also investigated. DPV analysis was performed using a single electrode under optimal conditions, and after the initial test, the electrode was stored. The ipa value for BPA decreased by 6.4% after a week (Figure S3D). This demonstrates that the Ti_3_C_2_T_x_ MXene/MHCs/GCE exhibits commendable stability. The results show that the Ti_3_C_2_T_x_ MXene/MHCs/GCE has good stability, reproducibility, and anti-interference performance.

### 2.8. Analytical Principle of the MXene/MHCs Composite

According to the above-mentioned results, a possible analytical principle of this electrochemical sensor is proposed (as shown in Scheme 1). Ti_3_C_2_T_x_ MXene, possessing a large specific surface area, exhibits a certain adsorption and enrichment effect towards BPA. Its excellent conductivity and accelerated electron transfer lead to an increase in peak current. In addition, the introduction of MHCs not only effectively increases the specific surface area of the electrode and avoids the problem of Ti_3_C_2_T_x_ MXene restacking, but also enhances the rich surface pores and good conductivity of MHCs, shortens the ion transport path, and accelerates electron transport. The DPV method is used to record the electrochemical response signal of BPA, and the current increase is used to accurately determine the content of BPA.

### 2.9. Actual Sample Detection of BPA

BPA was detected in tap water and laboratory wastewater. Firstly, a certain volume of sample solution and BPA standard solutions were sequentially added to PBS (pH = 4.0), and their corresponding ipa values were obtained (Table 2). Among them, Ti_3_C_2_T_x_ MXene/MHCs/GCE can accurately detect BPA in environmental samples, with actual sample recoveries ranging from 94.6% to 105.2% and an RSD of less than 4% (n = 3). In addition, the comparison between the obtained detection results and the standard method (spectral method) detection results is within the error range, indicating that the sensor can accurately detect the BPA content in actual samples.

## 3. Experiment

### 3.1. Materials and Reagents

Tetrapropoxysilane (TPOS), bisphenol A (BPA), hydroquinone (HQ), catechol (CC), and 4-nitrophenol (4-NP) were from Aa-laddin Reagent Co., Ltd., Shanghai, China. Resorcinol and formaldehyde were from Shanghai Macklin, Shanghai China. Ti_3_AlC_2_ was purchased from XFNANO (Nanjing, China). All the chemicals were of analytical grade and double-deionized water (18.25 MΩ) was chosen.

### 3.2. Apparatus

The electrochemical experiments were carried out in an electrolytic cell with a three-electrode system (CHI 650E electrochemical workstation, platinum wire counter electrode, Ag/AgCl reference electrode, and MXene/MHCs/GCE working electrode). The materials were characterized using a scanning electron microscope (Sigma HD ZEISS, Oberkochen, Germany), transmission electron microscopy (JEOL-JEM-2100F, Tokyo, Japan), and X-ray photoelectron spectrum (Ultima IV Hitachi, Tokyo, Japan).

### 3.3. Preparation of Ti_3_C_2_T_x_ MXene

Ti_3_C_2_T_x_ MXene nanosheets were synthesized by etching Ti_3_AlC_2_ using a LiF/HCl solution according to a reported method [21]. Specifically, 1.6 g of LiF and 20 mL of 9 M HCl were first mixed in a 50 mL polytetrafluoroethylene reactor under stirring, followed by reaction at 50 °C for 30 min. Then, 1 g of Ti_3_AlC_2_ was slowly added to the mixture in portions and allowed to react at 45 °C for 48 h. After the reaction, the product was repeatedly centrifuged and washed until the supernatant reached pH 6, and then collected via vacuum filtration. The resulting precipitate was further centrifuged until a viscous layer formed and the upper liquid appeared black. This precipitate was redispersed in 100 mL deionized water, transferred into a three-neck round-bottom flask, and purged with argon. The mixture was ultrasonicated at 5 °C for 1 h to achieve homogeneous dispersion. Finally, the dispersion was centrifuged for 0.5 h to obtain a dark green supernatant, which was then vacuum-pumped and freeze-dried to yield clay-like Ti_3_C_2_T_x_ MXenes.

### 3.4. Preparation of MHCs

MHCs were synthesized via a one-pot process as previously reported in the literature [25]. Briefly, Tetrapropoxysilane (TPOS) (3.46 mL, 12 mmol) was added to the solution containing ethanol (70 mL), H_2_O (10 mL), and NH_3_·H_2_O (3 mL, 25 wt %) under stirring at room temperature. After 15 min, resorcinol (0.4 g) and formaldehyde (0.56 mL, 37 wt %) were added to the solution and the system was continuously stirred for 24 h. The precipitates were separated by centrifugation (8000 rpm for 10 min), washed with water and ethanol three times, and dried at 50 °C overnight. After that, MHCs were obtained after carbonization at 700 °C under N_2_ for 5 h and silica was removed by NaOH (4 mol/L) for two days.

### 3.5. Preparation of MXene/MHCs

First, 30 mg of MHCs was dispersed in 10 mL of deionized water, and 20 mg of Ti_3_C_2_T_x_ MXenes was separately dispersed in 10 mL of ethanol, both yielding homogeneous solutions with a concentration of 3 mg/mL. The two solutions were then combined in equal volumes at a 1:1 concentration ratio. After stirring at room temperature for 1 h to achieve thorough mixing, the mixture was further treated with ultrasonication for 0.5 h to improve dispersion. Finally, the resulting product was dried under vacuum to obtain the MXene/MHCs composite.

### 3.6. Preparation of Modified Electrodes

Modified glassy carbon electrodes were prepared through a sequential procedure. First, the electrodes were polished using Al_2_O_3_ powder with varying particle sizes (1.0, 0.3, and 0.05 μm), followed by ultrasonic cleaning in ethanol and deionized water for 5 min each. After cleaning, the electrodes were dried under an infrared lamp for 3 min. Subsequently, a 2 mg/mL suspension of Ti_3_C_2_T_x_ MXene/MHCs was prepared and subjected to sonication for 1 h. Finally, 6 µL of the well-dispersed suspension was drop-cast onto the dried electrode surface and dried under an infrared lamp. The schematic diagram of the electrochemical sensor platform constructed using Ti_3_C_2_T_x_ MXene/MHCs/GCE for RFP detection is shown in Scheme 1.

### 3.7. Electrochemical Measurements

Electrochemical tests were performed using a CHI 650E electrochemical workstation (Shanghai Chenhua, Shanghai China). The reference electrode was Ag/AgCl, and the counter electrode was a platinum wire electrode. In total, 0.1 M phosphate buffer solution (PBS) was used as the supporting electrolyte. In cyclic voltammetry (CV), the scan rate was 100 mV·s^−1^, and the working window voltage was 0.2 V–1.0 V. In differential pulse voltammetry (DPV), the pulse amplitude was 0.05 V, the pulse period was 0.2 s, the enrichment time was 60 s, and the enrichment potential was 0 V. Electrochemical impedance tests were performed in a 0.1 M KCl solution containing 5.0 mM [Fe(CN)_6_]^3−/4−^ in the frequency range from 0.01 to 10^5^ Hz with an amplitude of 5 mV.

## 4. Conclusions

In this work, a high-performance electrochemical sensor for bisphenol A (BPA) was successfully constructed based on a Ti_3_C_2_T_x_ MXene/MHCs composite prepared via a simple ultrasonic method. The introduction of MHCs played a critical role in enhancing the sensor’s performance by effectively preventing the restacking of MXene sheets, thereby maximizing the accessible specific surface area. This synergistic combination created a material with rich surface porosity, excellent conductivity, and shortened ion transport pathways, collectively leading to accelerated electron transfer and electrochemical sensing capabilities.

The proposed sensor demonstrates significant potential for the development of low-cost, user-friendly, and efficient water quality monitoring platforms. Looking forward, this work provides a valuable research foundation and opens up several promising avenues for future development. Firstly, it highlights the great promise of MXene-based composite materials in the design of next-generation environmental sensors. Secondly, the sensor architecture could be integrated into portable or even wearable analytical devices, enabling the real-time on-site detection of BPA and other emerging contaminants. Ultimately, such advancements are crucial for strengthening environmental monitoring networks, informing regulatory decisions, and safeguarding public health against the risks posed by endocrine-disrupting chemicals.

## Figures and Tables

**Table 1:** Comparison of analytical performance of different BPA sensors.

Electrode	Method	Linear Range (μM)	LOD (μM)	Ref.
Cu-CDs/NH_2_-CNTs/GCE	DPV	0.5–160	0.13	[32]
PTB/AuNP/GCE	DPV	0.2–5.0	0.13	[33]
RGO-Ag/PLL/GCE	DPV	1–80	0.54	[34]
AgNPs-EG	DPV	5–100	0.23	[35]
SWCNT/GCE	Amperometry	10–100	7.3	[36]
PEDOT/GCE	CV	40–410	22	[37]
MIP PGE Sensor	DPV	1.5–7.5	0.1610	[38]
AuNP Platforms	DPV	0.002–1	0.0035	[39]
Ti_3_C_2_T_x_ MXene/MHCs/GCE	DPV	10–200	2.6	This work

**Table 2:** Analytical results for the BPA detection from real samples (n = 3).

	Added (μM)	Founded (μM)	Recovery (%)	RSD (%)	UV-Vis (μM)
tap water	0	0	--	--	
	20	18.92 ± 0.05	94.6	0.79	19.81 ± 0.23
	50	49.54 ± 0.10	99.1	3.5	52.36 ± 0.19
	100	105.19 ± 0.08	105.2	1.02	98.74 ± 0.07
laboratory wastewater	0	0	--	--	
	20	21.28 ± 0.09	106.4	0.36	20.57 ± 0.12
	50	50.96 ± 0.07	101.9	1.82	50.13 ± 0.25
	100	99.35 ± 0.05	99.4	2.1	101.62 ± 0.23

## Data Availability

The original contributions presented in this study are included in the article. Further inquiries can be directed to the corresponding author(s).

## Supplementary Materials

The following supporting information can be downloaded at: https://www.mdpi.com/article/10.3390/molecules30193992/s1, Scheme S1: The reasonable electrochemical reaction mechanism of BPA at MXene/MHCs/GCE. Figure S1: SEM images of (A) SiO_2_@SiO_2_@RF, (B) SiO_2_@SiO_2_@C. Figure S2: (A) The XRD patterns of MHCs, Ti_3_AlC_2_ and MXene. (B) The XPS survey of the SiO_2_@SiO_2_@C and MHCs. (C,D) N_2_ adsorption-desorption isotherms and pore size distribution of MHCs. Figure S3: (A) Influence of accumulation time on the oxidation peak current of 100 μM BPA. (B) Influence of accumulation potential on the oxidation peak current of 100 μM BPA. (C) The DPV response of BPA on modified electrodes of different proportion, MHCs/Ti_3_C_2_T_x_ MXene/GCE (1:1) (a), MHCs/Ti_3_C_2_T_x_ MXene/GCE (2:3) (b) and MHCs/Ti_3_C_2_T_x_ MXene/GCE (1:2) (c). (D) The change in ipa of MHCs/Ti_3_C_2_T_x_ MXene/GCE after a week of storage.
