# Supplementary material for: A Novel Electrochemical Sensor Based on Ti3C2Tx MXene/Mesoporous Hollow Carbon Sphere Hybrid to Detect Bisphenol A
Source: Molecules. 2025 Oct 5;30(19):3992. doi: 10.3390/molecules30193992 (PMC12526333; doi:10.3390/molecules30193992)
Supplement: Supplementary file 1 [file molecules-30-03992-s001.zip › molecules-3884643-supplementary.pdf]

Supplementary Material

A novel electrochemical sensor based on Ti_3C_2Tx MXene/mesoporous hollow carbon sphere hybrid to detection of Bisphenol A

Fei Cao[a], Qirong Zhou[a], Yanting Zhou[a], Yaqi Yang[a*], Li Zhang[a*], Yixi, Xie[a, b*]

[a] *College of Chemistry and Materials Engineering, Huaihua University, Huaihua 418000, People's Republic of China*

[b] *Key Laboratory for Green Organic Synthesis and Application of Hunan Province, Xiangtan University, Xiangtan 411105, People's Republic of China*

**Yaqi Yang, Ph.D., College of Chemistry and Materials Engineering, Huaihua University, Huaihua, China*

E-mail: yyq880@126.com

**Li Zhang, Ph.D., College of Chemistry and Materials Engineering, Huaihua University, Huaihua, China*

E-mail: zhangli@hhtc.edu.cn

** Yixi Xie, College of Chemistry, Xiangtan University, Xiangtan, China*

E-mail: xieyixige@xtu.edu.cn

Tel.: 86-731-58292060

List of Supplementary Tables and Figures:

Scheme S1. The reasonable electrochemical reaction mechanism of BPA at MXene/MHCs/GCE.

Figure S1. SEM images of (A) SiO_2@SiO_2@RF, (B) SiO_2@SiO_2@C.

Figure S2. (A) The XRD patterns of MHCs, Ti_3AlC_2 and MXene. (B) The XPS survey of the SiO_2@SiO_2@C and MHCs. (C-D) N_2 adsorption−desorption isotherms and pore size distribution of MHCs.

Figure S3. (A) Influence of accumulation time on the oxidation peak current of 100 μM BPA. (B) Influence of accumulation potential on the oxidation peak current of 100 μM BPA. (C) The DPV response of BPA on modified electrodes of different proportion, MHCs/Ti_3C_2Tx MXene/GCE (1:1) (a), MHCs/Ti_3C_2Tx MXene/GCE (2:3) (b) and MHCs/Ti_3C_2Tx MXene/GCE (1:2) (c). (D) The change in ipa of MHCs/Ti_3C_2Tx MXene/GCE after a week of storage.

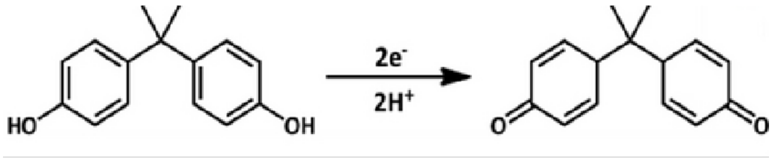

Scheme S1

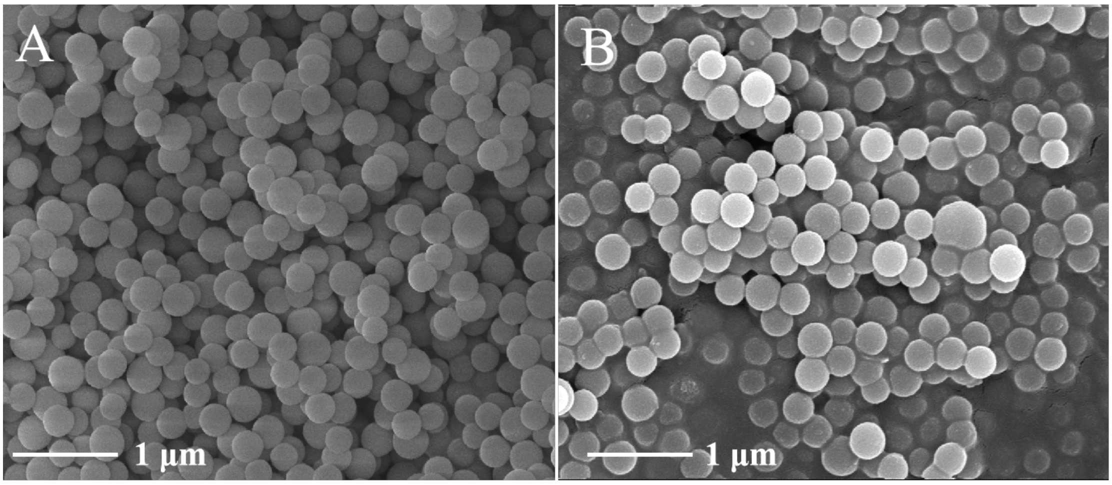

Figure S1

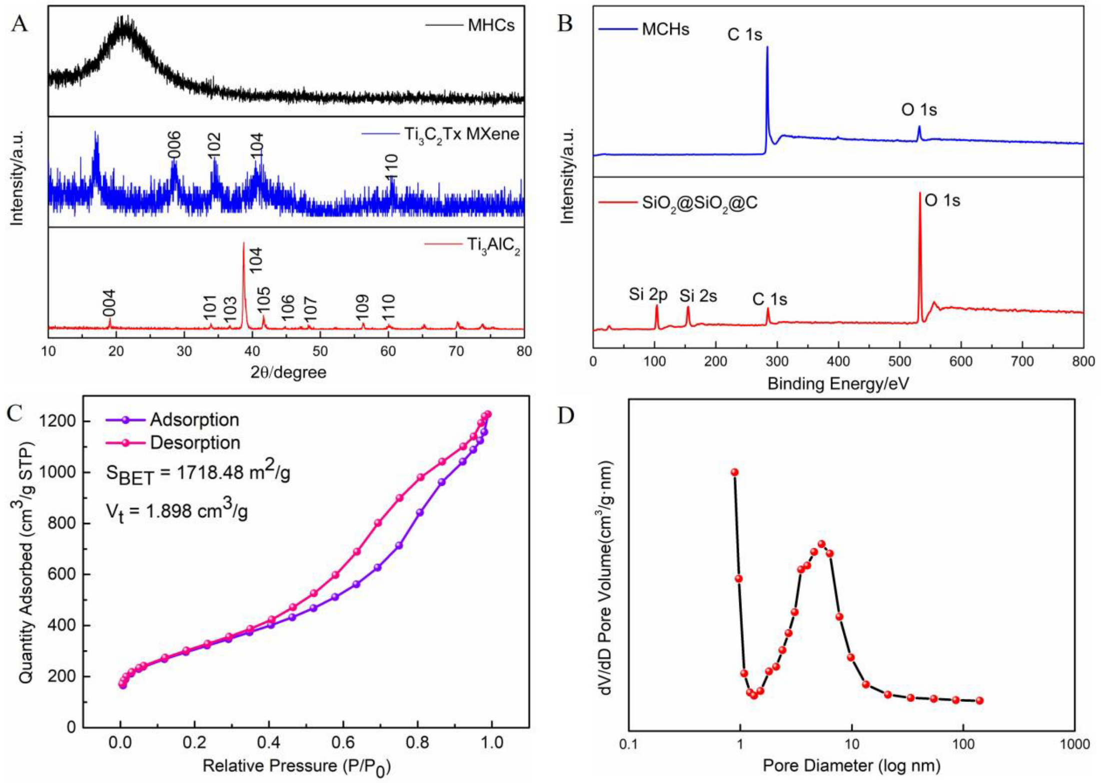

Figure S2

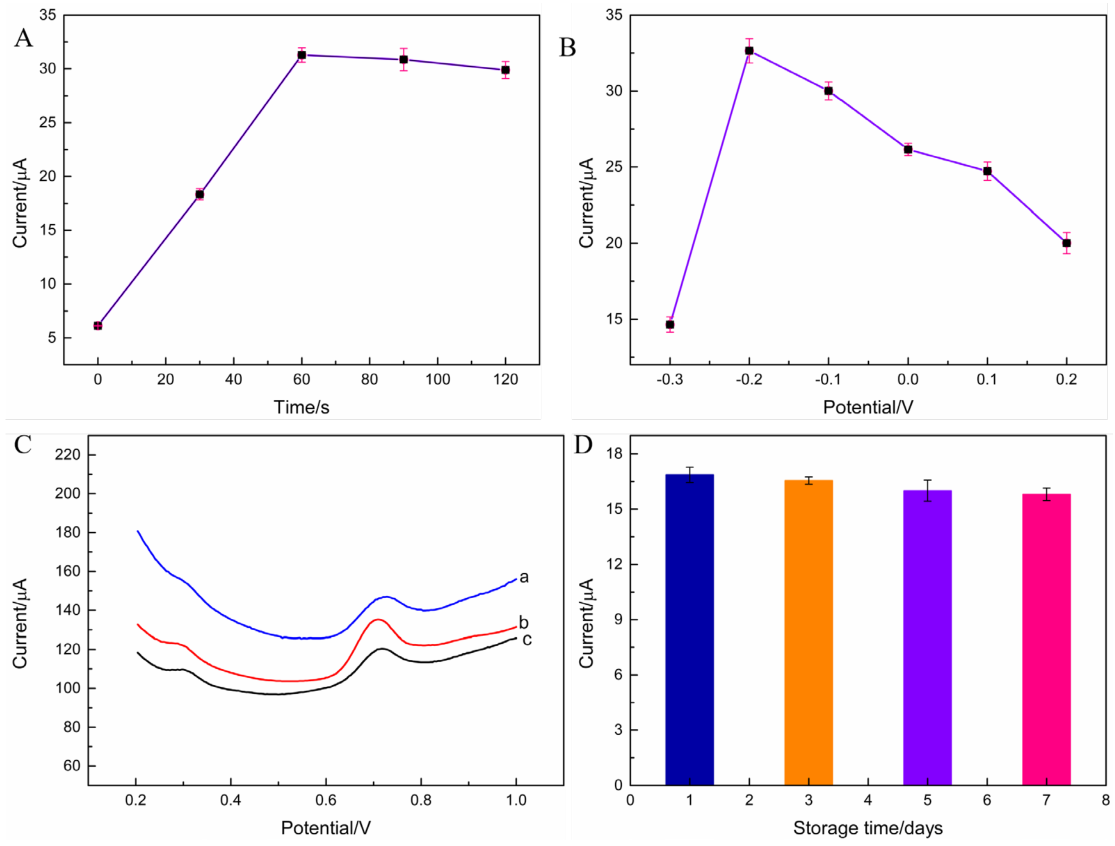

Figure S3
